# Abnormal expression of Nrf2 may play an important role in the pathogenesis and development of adenomyosis

**DOI:** 10.1371/journal.pone.0182773

**Published:** 2017-08-17

**Authors:** Ning Chen, Baoying Du, Hao Zhou, Fengxian Shen, Juan Li, Zhenwei Xie

**Affiliations:** 1 Department of Gynecology, Women’s Hospital, School of Medicine, Zhejiang University, Hangzhou, Zhejiang, P.R. China; 2 School of Medicine, Zhejiang University, Hangzhou, Zhejiang, P.R. China; West China Second Hospital, Sichuan University, CHINA

## Abstract

**Objective:**

To explore the expression level of Nrf2 in adenomyosis and study the mechanism of abnormal expression of Nrf2 in the pathogenesis of adenomyosis.

**Methods:**

Western blot, immunohistochemistry(IHC) and real time PCR were used to measure Nrf2 expression levels in tissue and cell samples. Knockdown and overexpression of Nrf2 were used to investigate the variation of migration ability of endometrial glandular cells as well as the regulatory mechanism.

**Results:**

Nrf2 protein levels were significantly higher in the eutopic and ectopic endometrial glands when compared with control cases using IHC and western blot methods. (p< 0.05). However, there was no statistical difference in Nrf2 mRNA expression levels between the adenomyosis and control groups. Using an agonist and Nrf2 siRNA, we regulated the Nrf2 protein levels of primary cultured endometrial glandular cells. With increased expression of Nrf2, cell scratch assay showed that the agonist-treated group migrated significantly faster than the control group, with MMP9 protein level markedly elevated. In contrast, Nrf2 siRNA-treated group migrated slower than the control group, with decreased expression of MMP9 protein. All of the scratching healing spaces and protein levels between the treated and control groups were statistically significant (*p*< 0.05).

**Conclusions:**

Abnormal expression of Nrf2 may play an important role in the pathogenesis and development of adenomyosis. Specified reduction of Nrf2 expression could prove to be a new therapeutic target in the clinical treatment of adenomyosis.

## Introduction

Adenomyosis is a common gynecological disorder, which is presented with a wide range of clinical presentations, such as heavy menstrual bleeding, dysmenorrhoea and infertility[1]. Although the age of onset is getting younger and incidence is rising in recent years, the mechanism of pathogenesis of adenomyosis is still incompletely understood. Both hysterectomy and many other kinds of conservative treatments can fail to completely relieve patient’s sufferings, and treatment options are especially limited for those planning for future pregnancies[2, 3].

As we know, there is an absence of submucousal layer between the basal endometrium and myometrium[4]. Iatrogenic injuries such as curettage, abortion, caesarean section and chronic inflammation may cause damage to the sub-endometrial myometrium. This is regarded as one of the key etiological factors of adenomyosis[5]. When damage happens, glandular cells may traverse a region of structural weakness and invade into the myometrium. Damage and inflammation bring forth increased oxygen uptake, leading to an increased release and accumulation of ROS at the site of damage[6].

Nrf2(Erythroid–E2-related factor 2) is one of the key nuclear transcription factor which defends against stress injuries through maintaining intracellular redox homeostasis and incorporatingan inflammatory response[7, 8]. Although accumulating evidences have indicated the protective effect of Nrf2 against oxidative stress, it’s been shown that sustained abnormal expression of Nrf2 promotes disease progression which is related with over-oxidative damage such as chronic kidney disease and various carcinomas[9–11].Nevertheless, Nrf2 expression levels in adenomyosis and its role in the pathogenesis remain unknown.

In this study, we aimed to investigate the Nrf2 expression in adenomyosis tissues and explored the mechanism by which Nrf2 promotes disease progression. Our results revealed that Nrf2 is highly expressed in adenomyosis tissues and gland cells. Furthermore, the role of Nrf2 in enhancing cell migration is supported by the fact that up-regulation of Nrf2 caused paralleled expression changes in the expression levels of matrix metallproteinase 9(MMP9), which is closely related with the degradation of extracellular matrix and cell migration[12], enabling enhanced cell migration.

## Materials and methods

### Case selection

A total of 40 endometria specimens were retrospectively selected from Women’s Hospital School of Medicine Zhejiang University with informed consent in accordance with the requirements of the Research Ethics Committee(see in S1 and S2 Tables). All the participants signed the written informed consents to participate in this study. There included 20 control cases which were pathological diagnosed as either cervical intraepithelial neoplasia III or carcinoma in situ(CIS) and 20 adenomyosis cases undergoing surgical therapy. Among all the studied cases, no malignancies further than CIS or those with personal cancer histories were included; Cases with myoma or using intrauterine device were excluded. Cases were reviewed using hematoxylin and eosin-stained slides independently by two pathologists.

### Nrf2 immunohistochemical (IHC) analysis

A rabbit monoclonal antibody (EP1808Y) which specifically reacts with human Nrf2 (IgG2) was purchased from Abcam, Inc. (Cambridge, MA). Immunohistological analysis of Nrf2 protein expression was performed as previously described. According to our studies, with sections of endometrial serous carcinoma with strong Nrf2 expression serving as positive control, and a criteria based on the positive score index we defined and applied previously[13]; only cases whose indices above 25 were regarded as positive.

### Cell isolation and culture

Eutopic endometrial tissues were collected from the uterine specimen with clinically diagnosed with adenomyosis. The entire process was under sterile conditions and tissues were transported to the laboratory on ice in DMEM (Dulbecco’s modified Eagle’s medium)/F-12 (Gibco, USA) with 10% fetal calf serum (FCS; Hyclone, Logan, UT, USA). The endometrial epithelial cells (EECs) were isolated according to reference ranges [14]. Briefly, endometrial tissue was digested with collagenase I and the subsequent suspension was filtered through 43 and 11 μm metal mesh to collect endometrial epithelial glands. The cells/epithelial fragments were collected and resuspended in DMEM/F-12 supplemented with 10% FCS and plated. HEEC were collected from the filter paper and further purified through selective adherence. Epithelial glands were serially replated (three times) in plastic culture dishes for 30 min, to allow adherence of contaminating stromal cells. Non-adherent cells/glands were transferred to 96- or 48-well plates and epithelial cells were allowed to grow out from glandular structures for 48 h. These cells were incubated at 37°C in a humidified atmosphere containing 5% CO2. Cells were identified to be epithelial in origin by morphology and immuno-chemical analysis with cytokeratin(CK) 8 and vimentin antibodies(S1 Fig).

### RNA isolation and qRT-PCR

Cell-free total RNA was extracted from cell lysates using the Trizol reagent (12183–555, Invitrogen, Carlsbad, CA, USA). cDNA was synthesized from 200 ng total RNA using oligo-dT primers (Invitrogen). qRT-PCR for each gene was carried out using a thermal cycler (Bio-Rad, Hercules, CA, USA) and amplification conditions were 40 cycles of 30s at 95°C, 3 s at 95°C, and 30 s at 60°C. Primers were synthesized by Bioneer (TaKaRa,Japan) and the primer sequences for NRF2 were, 5′- TCAGCGACGGAAAGAGTATGA -3′ and 5′- CCACTGGTTTCTGACTGGATGT -3′; and GAPDH, 5′- TGACTTCAACAGCGACACCCA -3′ and 5′CACCCTGTTGCTGTAGCCAAA -3′. Data were normalized to the expression of GAPDH, and PCR products were separated on a 1.2% agarose gel containing ethidium bromide, for quantification by densitometry using Image J software (National Institutes of Health, USA). Duplicated reactions were performed for each sample and the same experiment was repeated twice. http://dx.doi.org/10.17504/protocols.io.ixecfje

### Nrf2 regulation with t-BHQ or transfection of small interfering RNA

t-BHQ was purchased from Sigma (catalog number 112941). Nrf2 small interfering RNA (siRNA) (catalog number: SI03246614) and the control siRNA(catalog number:SI03650318) were purchased from Qiagen (Valencia, CA). Transient transfection of siRNA was performed using HiPerFect Transfection Reagent according to the manufacturer’s protocol (Qiagen). http://dx.doi.org/10.17504/protocols.io.iztcf6n

### Western blot analysis

Nrf2 monoclonal antibody was purchased from Abcam, Inc as mentioned above. The Keap1, MMP9 and dehydogenase (GAPDH)antibodies were purchased from Santa Cruz Biotechnology. Both cultured cells and tissues were subjected to immuno-blot analysis and proceeded as previously reported[15].http://dx.doi.org/10.17504/protocols.io.iy5cfy6

### t-BHQ pretreatment and cell scratch assay

After cells were cultivated in 24-well plates, change DMEM/F12medium after 24 h. When the spread rate reached 80%,t-BHQ was added into the treated group and make the final concentration as 50uM. After 24h, 10 μL pipette tips scratched in the plates in vertical directions. Recording of the scratching spaces under the microscope were made at 0, 24, and 48 hours.http://dx.doi.org/10.17504/protocols.io.izucf6w

### Statistical analysis

Results are expressed as mean ± SD. Comparison of IHC Nrf2 expression in different status of the endometrium was assessed by Chi-squares and Fisher's exact test when an expected cell value was 5 or less. Unpaired student t-tests were used to compare the means of two groups. Statistical tests were performed with SPSS 10.0. *P*<0.05 was considered to be significant.

## Results

### Immunostaining showed higher expression level of Nrf2 in adenomyosis

**Fig 1:**
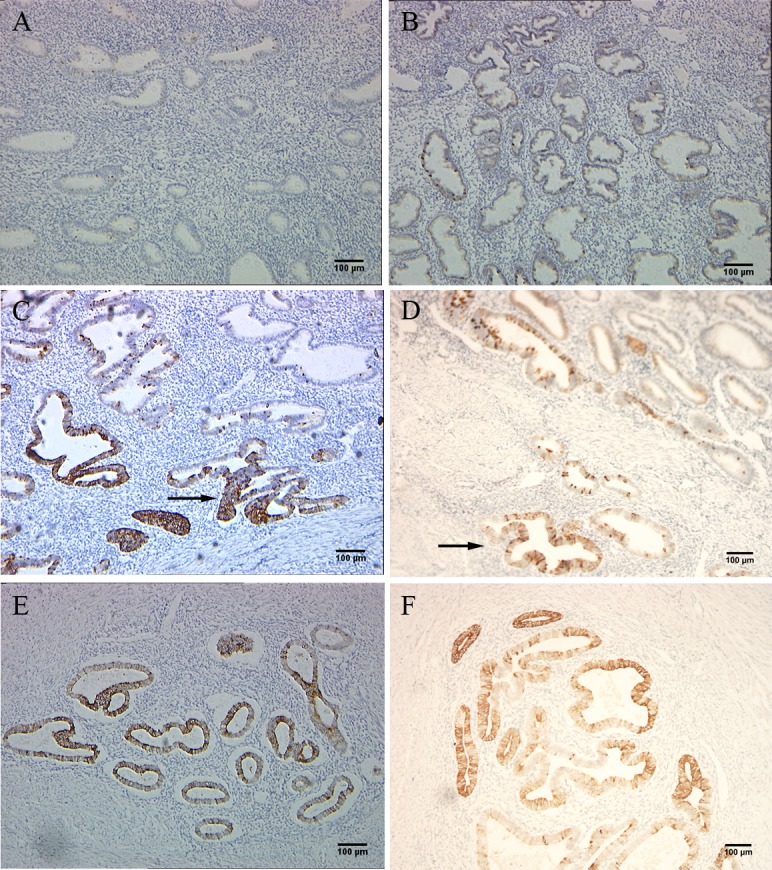
IHC staining shows that adenomyosis tissues expressed higher level of Nrf2 than control cases. A and B, proliferative and secretory phase endometria of control cases; C and D, eutopic endometrium of adenomyosis and with coexistent local ectopic focus in the latter image; E and F, ectopic endometrial foci of adenomyosis.

A total of 40cases including 20 adenomyosis and 20 benign control were examined. Either case or control group presented 10 proliferative and 10 secretory endometria tissue. H&E slides were reviewed to confirm the pathological diagnosis. Nrf2 expression was scored as positive or negative for each case using score index(case index≥25 was defined as positive) according to the criteria defined and applied previously. Among the 20 adenomyosis cases, 15 (75%) were positive for Nrf2 expression both in eutopic and ectopic endometria. Which should be mentioned is, all the cases with positive eutopic endometria had a clear ectopic expression of Nrf2. In contrast, Nrf2 was only expressed in 3 of 20 (15%) benign cases. All the staining data of 40 cases were shown in S3 Table. For all the positive staining areas, Nrf2 was expressed only in endometrial glandular cells, and no expression in stromal cells. The difference in Nrf2 expression was statistically significant between adenomyosis and the control cases (*P*< 0.05). Furthermore, we found that the closer to the basal layer, the more concentrated the Nrf2 staining occurred. Representative images are shown (Fig 1), whereas positive case distribution was summarized in Table 1.

**Table 1:** Comparison of Nrf2 protein levels in benign and adenomyosis tissues.

	No. of cases	Positive(+)	Percentage(%)
Normal	20	3	15
Eutopic	20	15	75*
Ectopic	20	15	75*
Total	60	33	55.6

p<0.0.1compared with benign group.

### Differentiation of protein expression in specimen by western blot

**Fig 2:**
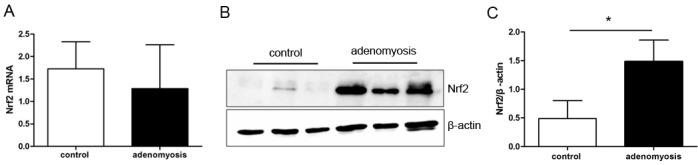
Adenomyosis tissues showed higher expression of Nrf2. A, mRNA level of Nrf2 was compared between benign and adenomyosis tissues using real time PT-PCR. The data presented were normalized to GAPDH; B, The protein level of Nrf2 was compared between control and adenomyosis tissues; C, Intensity of the western blot bands was quantified and compared between groups.

Next, we examined Nrf2 expression in 40 endometria tissue specimens, half of which were those with adenomyosis and the other half with CIN III or cervical CIS. As shown in Fig 2, the Nrf2 protein was highly expressed in endometria with adenomyosis. In contrast, marginal expression of Nrf2 was detected in control groups(For the other cases not showed, please find in S2 Fig). The cases with highly expressed Nrf2 detected by western blot proved to have strong reactivity to Nrf2 in immuno-staining. Therefore, the result of IHC and western blot were consistent with each other.

### Measuring Nrf2 mRNA expression in endometrial tissues

We also used RT-PCR and real-time PCR to measure and compare the expression levels of endometrial tissues between control and adenomyosis cases. No difference in Nrf2 mRNA transcription level was observed in adenomyosis endometria compared with control group. This is in agreement with previous experimental results in endometrial cancer research, which suggest that Nrf2 protein expression levels may be more strongly affected by the ubiquitination degradation mediated by Keap1, than by transcription and translation.[15].

### Up-regulation of Nrf2 enhanced the migration ability of endometrial glandular cells

**Fig 3:**
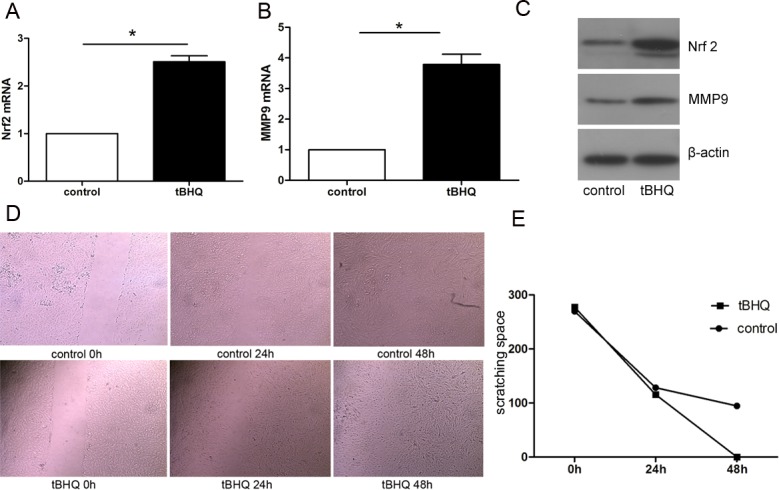
Up-regulation of Nrf2 in normal endometrial glandular cells was directly correlated with increased expression of MMP9, and thus caused significantly stronger migration ability of primary cultured glandular cells. A and B, the mRNA levels of Nrf2 and MMP9 in control and tBHQ groups were measured; C, higher protein levels of Nrf2 and MMP9 in tBHQ group is shown by western blot; D, glandular cells were undergone scratch test, while the wound healing changes were recorded between two groups at 0h,24h,48h; E, The variation between groups is shown in the line chart (p<0.05).

To further explore the role of Nrf2 in the pathogenesis of adenomyosis, tBHQ was added into the epithelial cells culture system before the scratch assay to up-regulate the Nrf2 expression. As shown in Fig 3, the protein levels of Nrf2 were increased significantly in the pretreated group compared with the control group. Accordingly, the protein levels of MMP9 raised markedly. 48 h after scratching, the tBHQ pretreated cells were found to migrate significantly faster than the control group. The scratch spacing at the 0h, 24h, 48h, time points were calculated and are shown in Fig 3. The difference of wound healing speed between groups was statistically significant (p<0.05).

### Decreasing Nrf2 expression suppressed the transference of endometrial glandular cells

**Fig 4:**
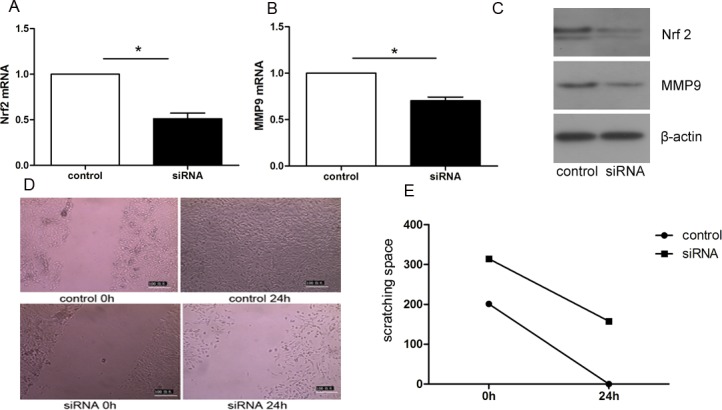
Transient knockdown of Nrf2 expression by Nrf2 siRNA decreased the migration ability of primary cultured glandular cells, with reduced MMP9 mRNA and protein levels. A and B, the mRNA levels of Nrf2 and MMP9 in control and Nrf2 siRNA groups were measured; C, lower protein levels of Nrf2 and MMP9 in Nrf2 siRNA group is shown by western blot; D, glandular cells were undergone scratch test, while the wound healing changes were recorded between two groups at 0h,24h; E, The variation between groups is shown in the line chart (p<0.05).

As shown in Fig 4, after transient transfection of Nrf2 siRNA, Nrf2 expression was examined at both the RNA and protein levels. With an apparent decline of Nrf2 expression, MMP9 mRNA and protein levels were consequently reduced to different extents. In the scratch healing assay, with low levels of Nrf2 and MMP9, endometrial glandular cells transferred slowly and even could not maintain normal differentiation and proliferation. The test was repeated three times and the scratch spacings were quantified and shown in Fig 4.

## Discussion

Although being defined as a benign disease, adenomyosis showed noticeably malignant biological behavior, which caused diffuse lesions in the uterine myometrium[16]. Clinically, the age of patientsare is getting younger and younger. Some patients ranged from 30s-40s, were compelled to go under operation due to severe dysmenorrhea, anemia or hypermenorrhea[17, 18]. Therefore, exploring the pathogenesis is helpful to define new therapeutic targets and develop novel therapies.

As one of the most important cell transcription factors which regulate anti-oxidative downstream genes, abnormal expression level of Nrf2 has been shown to be closely connected with the development of many diseases[10, 19–22].

In this study, we first investigated the expression state of Nrf2 in adenomyosis. We determined that Nrf2 was marginally expressed in normal endometrium; while more than70% of adenomyosis cases were positive for high expression level of Nrf2. Upon further observation, glandular cells nearer to the myometrium showed stronger staining intensity. This phenomenon suggests that enhanced Nrf2 expression may be a key promoting element for the glandular cells transferring to the myometrium. However, the result displaying staining scores of Nrf2 in eutopic and ectopic endometrium had no statistical difference. Examination of RNA and protein level using clinical endometrial tissue showed that Nrf2 protein was apparently higher in adenomyosis than in normal controls, while the fact that differences of Nrf2 RNA levels between two groups had no statistical significance. Consistently, subsequent research showed that Nrf2 RNA levels had no obvious differences between control and tBHQ treated groups, although tBHQ induced up-regulated Nrf2 protein level. These phenomena proved our previous deduction that regulation of Nrf2 expression was mainly reduced from the degradation module caused by expression alteration of Keap1, not the gene translation and protein synthesis modules of Nrf2[15].

To further investigate the possible role of Nrf2 in the pathogenesis of adenomyosis, we used primary cultured endometrial glandular cells derived from the endometrium tissue of adenomyosis patients. After having successfully separated from mixed cells, glandular cells were identified by immuno-staining results of cytokeratin 8 positive and vimentin1 negative. Interestingly, after being cultured for 2-3days in vitro, we found that about 60%-70% cells had trans-differentiated to mesenchymal cells in the in-vitro environment. Anyway, the cause of this phenomenon is worth further investigation. We subsequently showed that, one of the representative proteins which functions to degrade extracellular matrix, MMP9, was expressed in tandem with Nrf2, alternating between high and low expression under the influence of tBHQ or Nrf2 siRNA. Correspondingly, cell scratching test indicated that cell migration ability enhanced notably with the increasing expression of Nrf2, and vice versa, diminishing while Nrf2 decreased. This indicates that alterations of Nrf2 could trigger changes in the ability of endometrial glands to migrate in adenomyosis, possibly through the regulation of MMP9 expression.

## Conclusion

In conclusion, our study showed that Nrf2 is overexpressed in adenomyosis tissues and glandular cells when compared to normal controls, suggesting that Nrf2 plays a role in the pathogenesis and development of adenomyosis. Studies using Nrf2 siRNA further indicated that knockdown of Nrf2 significantly decreased the ability of glandular cells to migrate from the basal layer to myometrium, suggesting that specifically decreasing Nrf2 expression may be a new therapeutic target for the clinical treatment of adenomyosis.

## Supporting information

S1 Table The opinion of medical ethics committee of Women’s Hospital, School of Medicine, Zhejiang University(Chinese vesion). (PDF)

S2 Table The opinion of medical ethics committee of Women’s Hospital, School of Medicine, Zhejiang University(English vesion). (PDF)

S3 Table The positive staining index(PSI) of 40 enrolled cases. The first table is about the PSI data of 20 normal endometria cases which were grouped into proliferative and secretory phases; the second table is the PSI data of 20 adenomyosis cases and the eutopic endometria were separately evaluated from ectopic foca.
(PDF)

S1 Fig Identification of endometrial glandular cells. Fig A is the staining result with CK8; Fig B is the staining result with vimentin.
(TIF)

S2 Fig The western blot results of the 34 cases other than the 6 showed in the manuscript. A1-4, B1-4, C1-2, D1-4, E1-3 are normal control cases respectively compared with A5-8, B5-8, C1-2, D5-8, E4-6. The bands concentrations were semiquantitatively evaluated and the data was compared between groups.
(TIF)
