# Supplementary material for: Abnormal expression of Nrf2 may play an important role in the pathogenesis and development of adenomyosis
Source: PLoS One. 2017 Aug 17;12(8):e0182773. doi: 10.1371/journal.pone.0182773 (PMC5560740; doi:10.1371/journal.pone.0182773)
Supplement: S1 Table — (PDF) [file pone.0182773.s001.pdf]

20

## 浙江大学医学院附属妇产科医院

# 医学伦理委员会意见书

<table>
<tr><td>项目类型</td><td colspan="5">1、临床科研　2、临床基础科研　3、药物验证　4、医疗仪器试剂验证<br>5、新技术开展</td></tr>
<tr><td>项目名称</td><td colspan="2">雌激素调控的Nrf2-ARE通路在子宫腺肌病腺上皮细胞侵袭迁移中的作用及机制研究</td><td>伦理批准号</td><td colspan="2">20160108</td></tr>
<tr><td>项目负责人</td><td>谢臻蔚</td><td>职称</td><td>主任医师</td><td>所在科室</td><td>妇四科</td></tr>
<tr><td>项目参与者</td><td colspan="5">陈宁、沈凤贤、王彩琴</td></tr>
<tr><td>项目实施的伦理学问题</td><td colspan="5">1、对受试者可能带来的心理、生理、生活、经济及其他损害和不利影响，防范和补救对策。<br>2、对受试者可能带来的近、远期潜在危险及其防范<br>3、其它伦理学问题（包括受试者个人、家庭、社会、人类等方面）<br><br>本研究项目拟收集宫颈癌 IA 期病人及子宫腺肌病人子宫切除术中的内膜组织，量约 1-2g，不影响患者的临床及病理诊断，不对患者的经济产生不利影响，对参加本课题的受试者的心理、生理、生活无明显不利影响及损害。课题研究方案对受试者近远期无明显潜在危险。</td></tr>
</table>

20

| | |
|---|---|
| 报送材料 | 1、知情同意书<br>2、研究项目书<br>3、<br>4、<br>5、<br>6、<br>7、<br>8、<br>9、<br>10、<br>申请者签名： |
| 伦理委员会意见 | 是否符合简易程序：<br>1、是 ✓<br>2、否<br>预审专家（签名）：<br>审查结果：<br>1、✓同意<br>2、修改后同意<br>3、修改后重审<br>4、不同意<br>伦理委员会主任（签名）：<br>[stamp: …江大学医学院附属妇产科医院 医学伦理委员会] |
