# Supplementary material for: Abnormal expression of Nrf2 may play an important role in the pathogenesis and development of adenomyosis
Source: PLoS One. 2017 Aug 17;12(8):e0182773. doi: 10.1371/journal.pone.0182773 (PMC5560740; doi:10.1371/journal.pone.0182773)
Supplement: S2 Table — (PDF) [file pone.0182773.s002.pdf]

Women's Hospital,Zhejiang University School of Medicine

Medical Ethics committee's opinion

<table>
<tr><td>Project Type</td><td colspan="5">1.Clinical Research 2.Clinical basic research 3.Drug verification
4.Validation of medical instrument and reagent 5.Development of New technology</td></tr>
<tr><td>Project name</td><td colspan="2">The role of Nrf2-ARE pathway regulated by estrogen in pathogenesis of adenomyosis and the mechanism study</td><td>Ethical approval no.</td><td colspan="2">20160109</td></tr>
<tr><td>Project leader</td><td>Zhenwei Xie</td><td>Profession</td><td>Chief physician</td><td>Department</td><td>Gynecology</td></tr>
<tr><td>Project participants</td><td colspan="5">Ning Chen, Fengxian Shen, Caiqin Wang</td></tr>
<tr><td>The Ethics problem of the project practice</td><td colspan="5">1. We should consider possible adverse effects on the subjects in physical, mental, and economical aspects as well as the corresponding measures of prevention and remedy.
2. We should prevent the immediate and long term potential hazard to the subjects.
3. Other ethics problem(including individual subject, family, society and human being)

The study will collected the endometrium tissues of whom underwent hysterectomy because of adenomyosis and CIN III and cervical carcinoma in situ . The specimen obtained should receive the patient's informed consent. The specimen collection will not affect the clinical and pathological diagnosis. The project has no side effect on physiplogy, life quality. Besides, no additional economic burden will be imposed on the patients. There will be no recent and long-term potential risk.</td></tr>
</table>

The submitted materials	1. Informed consents (All the participants signed the written informed consents to participate in this study)<br>2. Research project<br><br>Applicant : Zhenwei Xie
Medical Ethics committee's opinion	Result<br>Agreement ✓<br>Agreed provisionally (agreement after modified according the opinion)<br>Postpone to next meeting (The decision can't be made because the complementary material is needed)<br>Not agreement (More than two thirds of the committee against it)<br><br>Director of the ethics committee: Ruijin Wu
