# Supplementary material for: Abnormal expression of Nrf2 may play an important role in the pathogenesis and development of adenomyosis
Source: PLoS One. 2017 Aug 17;12(8):e0182773. doi: 10.1371/journal.pone.0182773 (PMC5560740; doi:10.1371/journal.pone.0182773)
Supplement: S3 Table — The first table is about the PSI data of 20 normal endometria cases which were grouped into proliferative and secretory phases; the second table is the PSI data of 20 adenomyosis cases and the eutopic endometria were separately evaluated from ectopic foca. (PDF) [file pone.0182773.s003.pdf]

Table 1 The positive staining index(PSI) of normal endometria (case1-20)

	1	2	3	4	5	6	7	8	9	10
Proliferative phase	30%×1	5%×1	0%×0	15%×1	10%×1	5%×1+3%×2	10%×2	10%×1+5%×2	20%×1+5%×2	10%×1
Secretory phase	0%×0	15%×1	20%×1	18%×1	10%×1	5%×2	30%×1+10%×2	0%×0	10%×2	15%×1

Table 2 The positive staining index(PSI) of eutopic and ectopic endometrial of adenomyosis cases（case1-20）

	1	2	3	4	5	6	7	8	9	10
Eutopic endometria	15%×2	10%×1 +30%×2	0%×0	0%×0	20%×1 +5%×2	5%×2	30%×2	5%×2	30%×1 +10%×2	25%×1
Ectopic endometria	0%×0	60%×2	20%×1 +5%×2	10%×1	40%×1 +20%×2	20%×1 +5%×2	20%×1 +40%×2	5%×2	60%×1 +30%×2	40%×1
	11	12	13	14	15	16	17	18	19	20
Eutopic endometria	20%×1 +5%×2	30%×1	15%×1	10%×1 +20%×2	15%×2	40%×1	30%×2	15%×2	45%×1	35%×1
Ectopic endometria	30%×2	25%×1 +15%×2	10%×2	30%×1	20%×2	20%×2	30%×1 +20%×2	5%×2	50%×2	25%×1 +20%×2
