# Supplementary figures and images for: Abnormal expression of Nrf2 may play an important role in the pathogenesis and development of adenomyosis
Source: PLoS One. 2017 Aug 17;12(8):e0182773. doi: 10.1371/journal.pone.0182773 (PMC5560740; doi:10.1371/journal.pone.0182773)

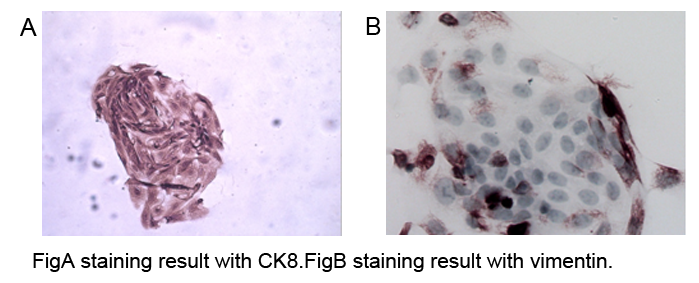

Supplement: S1 Fig — Fig A is the staining result with CK8; Fig B is the staining result with vimentin. (TIF) [file pone.0182773.s004.tif]

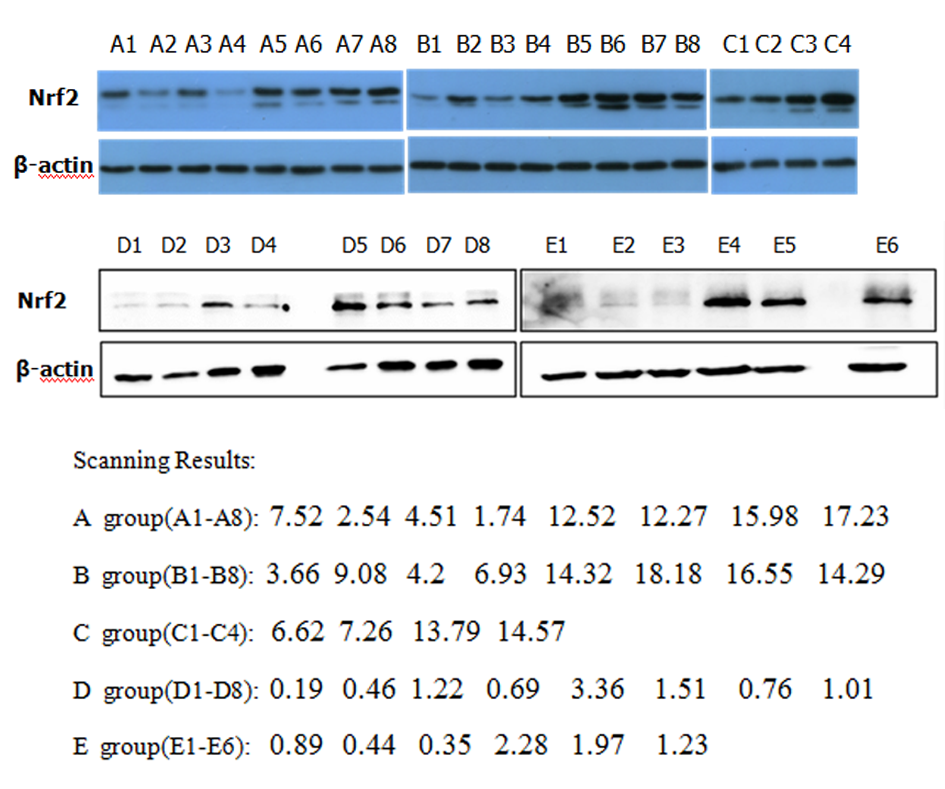

Supplement: S2 Fig — A1-4, B1-4, C1-2, D1-4, E1-3 are normal control cases respectively compared with A5-8, B5-8, C1-2, D5-8, E4-6. The bands concentrations were semiquantitatively evaluated and the data was compared between groups. (TIF) [file pone.0182773.s005.tif]
